# Decoding the anti-cancer potential of Pexidartinib (PLX3397), a Fms-like tyrosine kinase 3 inhibitor, using next-generation knowledge discovery methods

**DOI:** 10.6026/973206300200460

**Published:** 2024-05-31

**Authors:** Roaa Mahdi Alreemi

**Affiliations:** 1 Department of Biochemistry, College of Science, University of Jeddah, Jeddah, Saudi Arabia

**Keywords:** AML, FLT3, PLX3397, targeted therapy, receptor tyrosine kinase

## Abstract

Acute Myeloid Leukemia (AML) is a complex hematologic malignancy characterized by the rapid proliferation of abnormal myeloid precursor cells. The FMS-like tyrosine kinase 3 (FLT3), a receptor tyrosine kinase, plays a pivotal role in regulating cell survival, proliferation, and differentiation within the hematopoietic system. Mutations in FLT3, particularly internal tandem duplications (ITDs) and point mutations within the tyrosine kinase domain (TKD), are prevalent in AML and are associated with poor prognosis and increased risk of relapse. The development of targeted therapies has revolutionized the landscape of cancer treatment by focusing on the inhibition of kinase signalling. Small-molecule inhibitors designed to selectively target receptor tyrosine kinases, such as PLX3397, have shown promising results in preclinical studies and early phase clinical trials. PLX3397 exerts its inhibitory effects by targeting CSF1R and KIT, leading to the disruption of receptor tyrosine kinase signalling cascades, suppression of leukemic cell growth, and induction of apoptosis. This study emphasizes the significance of FLT3 as a receptor tyrosine kinase as a therapeutic target for PLX3397. After evaluating the usefulness of PLX3397 as an enzyme inhibitor using ADMET prediction, PLX3397 was prepared for molecular docking in the FLT3 crystal structure (PDB: 4XUF). A molecular dynamics simulation was performed on PLX3397 to evaluate its binding affinity and protein stability in a simulated physiological environment. In conclusion, targeting FLT3 as a receptor tyrosine kinase with PLX3397 represents a promising therapeutic strategy for improving outcomes in patients with FLT3-mutated AML. Further clinical investigations are warranted to validate the efficacy and safety of PLX3397 and to optimize treatment strategies for AML patients based on the FLT3 mutational status.

## Background:

Acute myeloid leukemia (AML) is a cancer that affects the blood and bone marrow. It involves the rapid growth of abnormal myeloid cells, which are a type of white blood cells responsible for fighting infections. AML accounts for approximately 1% of all cancer cases worldwide [1]. Although AML is one of the less common types of cancer compared to other types, its treatment presents several challenges due to the complex nature of the disease and its heterogeneity. Some key challenges in the treatment of AML include high relapse rates, drug resistance, limited treatment options for elderly patients, and the toxicity of intensive chemotherapy [2]. AML is characterized by genetic and molecular abnormalities that contribute to its pathogenesis and clinical heterogeneity. Many genes and genetic alterations are commonly associated with AML including FLT3 (FMS-like tyrosine kinase 3). Chromosome 13q12 carries the FLT3 gene, which is commonly referred to as FMS-like tyrosine kinase 3. This gene encodes FLT3, a receptor tyrosine kinase (RTK). The protein encoded by FLT3 is comprised of three domains. The extracellular domain is responsible for ligand binding, the Trans membrane domain anchors the protein to the cell membrane, and the intracellular domain contains tyrosine kinase activity necessary for signal transduction [3]. The FLT3 protein is essential for controlling several biological functions, especially hematopoiesis, to generate new blood cells. It contributes to the proliferation and maintenance of hematopoietic progenitor cells (HPCs) and stem cells (HSCs) in the bone marrow. Furthermore, FLT3 signalling pathways control functions such as differentiation, survival, and cell division [4]. Mutations in FLT3 have the potential to activate FLT3 signalling, which may promote abnormal cell reproduction and survival. Acute myeloid leukemia (AML) is most commonly associated with two types of FLT3 mutations: point mutations in the tyrosine kinase domain (TKD) and internal tandem duplications associated with the juxta membrane domain. AML patients with these mutations typically have poor prognosis [2], [3], [5]. Targeted therapy is a type of cancer treatment that specifically targets molecules or pathways involved in the development and survival of cancerous cells while minimizing damage to normal cells. In contrast to conventional chemotherapy, which frequently targets rapidly dividing cells randomly, targeted therapy intends to interfere with specific molecular targets that are exclusive to cancer cells or are essential for their proliferation and viability. Owing to the significance of FLT3 in the initiation and progression of malignancy, the FLT3 gene is considered an attractive therapeutic target, especially in the treatment of AML patients with FLT3 mutations. FLT3 inhibitors work by targeting the FLT3 receptor tyrosine kinase protein, inhibiting its activity, and thereby slowing the growth and division of leukemia cells. These inhibitors can help reduce AML progression and improve patient outcomes [6]. Examples of FLT3 inhibitors include midostaurin, gilteritinib, sorafenib, quizartinib, crenolanib, ponatinib, and FF-10101, which have been approved for the treatment of AML with FLT3 mutations. These targeted therapies are used alone or in combination with standard chemotherapies [2], [3], [6], [7] Pexidartinib (PLX3397) is a novel small chemical molecule that was initially investigated as a potential treatment for tenosynovial giant cell tumor (TGCT), a rare type of tumor, and received FDA approval in 2019 for this indication. It mainly targets two receptor tyrosine kinases (RTKs), colony-stimulating factor 1 receptor (CSF1R) and c-KIT, which are linked to several malignancies and inflammatory disorders [8]. By inhibiting these kinases, PLX3397 can block the growth of various cancers [9]. Although PLX3397 primarily targets CSF1R and c-KIT, it can also inhibit the expression of other genes. According to the Swiss Target Prediction and SuperPred databases, FLT3 was the top target of this inhibitor. Hence, we document the decoding the anti-cancer potential of Pexidartinib (PLX3397), a Fms-like tyrosine kinase 3 inhibitor, using next-generation knowledge discovery methods.

## Methodology:

## Target Prediction:

The web servers SuperPred and Swiss Target Prediction are knowledge-based methods that use machine-learning models to predict the targets of the investigated compound.

## WebGestalt Analysis:

WebGestalt database was selected for Gene Ontology (GO) terms, including biological process (GO-BP) and molecular function (GO-MF). The reference list for each analysis comprised all mapped gene symbols from the chosen platform genome, Homo sapiens as the species, and the parameters for the enrichment analysis were set at a minimum of 5 and a maximum of 2000 IDs in the category, a false discovery rate (FDR) of P ≤ 0.05, computed using the Benjamini-Hochberg (BH) method, and the significance level of the top 30, as previously described.

## Protein Preparation:

The crystal structure of receptor tyrosine kinase FLT3 in complex with the inhibitor quizartinib was obtained from the Protein Data Bank (PDB: 4XUF) and prepared using Schrödinger's Protein Preparation Wizard. Preparation includes the addition of missing hydrogen atoms to residues, correction of metal ionization states, and removal of water molecules > 5 Å from protein residues. Using Epik, the protonation state of the residues was generated and the formal charge on the metal ions was adjusted. After removing the extra protein subunit of the multi-subunit protein and additional ligands, protein processing was refined by predicting the pKa of the ionizable residues using PROPKA. Finally, the restrained minimization of the protein was performed using the OPLS4 force field.

## Ligand Preparation:

PLX3397 and co-crystallized ligands were prepared for docking using Schrödinger's LigPrep tool. This tool converted the 2D structures to 3D structures and energy-minimized those using the OPLS3 force field. After adding hydrogens, all possible ionization states and tautomeric forms were created at a pH range of 7.0 _ 2.0 by Epik; the desalting option was also chosen. The hydrogen bonds were optimized by predicting the pKa of the ionizable groups using PROPKA.

## Grid Generation and Molecular Docking:

The co-crystallized structure of AC220 was used as the reference to define the grid box. Glide's Receptor Grid Generation tool was used to determine the binding pocket. The grid box dimensions were set to 10 Å in each of the X, Y, and Z directions using the default settings without any modifications. Docking was then performed using the Schrödinger suite "Ligand Docking" tool. The selected docking protocol was extra precision (XP), and the ligand sampling method was flexible. All other settings were the default.

## Molecular dynamics (MD) simulation:

MD simulations were performed using Desmond software in the Schrödinger suite, limiting the run to include only the isomers with the highest docking scores. To ensure accurate results, the protein-ligand complex was immersed in a solvated system created by placing the complex in an orthorhombic water box that extended 10 Å beyond the atoms in the complex. Na + and Cl - counterions were added to neutralize the system. The simulation was set to continue for 100 ns, maintaining a constant temperature of 300 K and pressure of 1.01325 bars.

## ADMET Property Prediction:

ADMET prediction of PLX3397 isomers was performed using the QikProp module of the Schrödinger suite. The descriptors: molecular weight (mol_MW), drug-likeness (#Stars), dipole moment (dipole), total solvent accessible surface area (SASA), number of hydrogen bond donors and acceptors (donorHB and acceptHB), predicted octanol-water partitioning (QPlogPo/w), predicted aqueous solubility (QPlogS), estimated binding to human serum albumin (QPlogKhsa), number of the possible metabolites (# metab), predicted blood-brain partitioning (QPlogBB), percentage of human oral absorption, predicted IC50 for inhibiting HERG-K+ channels (QPogHERG), central nervous system activity (CNS), and number of reactive functional groups present (#rtvFG), were predicted for PLX3397 isomers. The predicted values were compared to the range observed for 95% of known drugs.

## Results and Discussion:

## Drug Targets:

**Figure 1:**
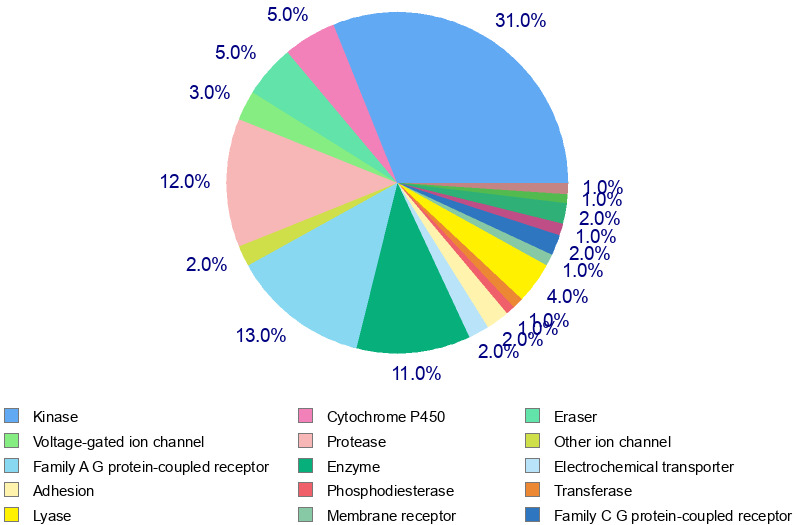
Pie chart of target classes based on the SwissTargetPrediction tool.

**Figure 2:**
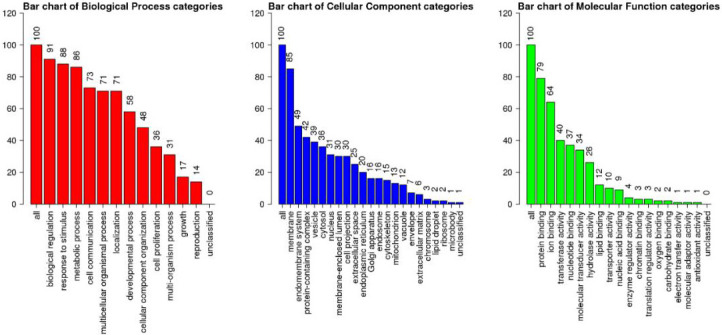
Gene ontology (GO) Slim Summary of the differentially expressed genes (DEGs) used in WebGestalt analysis for biological processes (red), cellular components (blue), and molecular functions (green)

PLX3397 targets several classes of proteins based on the data obtained from Swiss Target Prediction and Superpred (Table 1 and Table 2). SwissTargetPrediction for the ligand molecule PLX3397 has shown probable attachment to the biological system. The prediction report revealed the top probable targets given in the pie chart. The pie-chart shows that 31% of the total targets among the feasible targets are kinases, followed by family A G protein-coupled receptors (13%) and proteases (12%) (Figure 1). PLX3379 interferes with many biological processes including regulation, response to stimulation, and metabolism. It targets many cellular components, particularly the membrane, the endomembrane system, and protein-containing complexes. Furthermore, many molecular functions, including protein binding, ion binding, and transfer activities, are affected by PLX3397 (Figure 2).

## Gene Ontology biological process:

**Figure 3:**
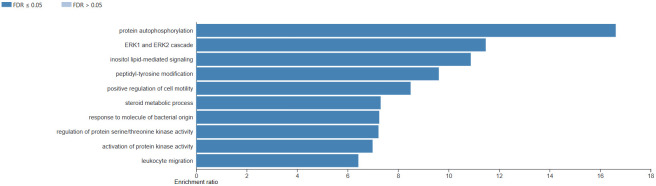
The bar shows the results of the analysis of the biological processes affected by PLX3397

PLX3397 may interfere with many biological processes, such as protein autophosphorylation, ERK1 and ERK2 cascades, and inositol lipid-mediated signaling (Figure 3). The ERK1/2 (Extracellular Signal-Regulated Kinase 1/2) cascade, also known as the MAPK/ERK pathway, plays a critical role in signaling cascades, and transmits extracellular signals to intracellular targets. This pathway controls signaling involved in various cellular processes, including cell proliferation, differentiation, survival, and migration in both healthy and pathological cases [10]. The activation of ERK1/2 has an important role in the development and progression of cancer. In cancer cells, mutations or alterations in upstream signaling molecules such as receptor tyrosine kinases (RTKs), Ras proteins, or Raf kinases can lead to constitutive activation of the ERK1/2 pathway. This prolonged activation of ERK1/2 promotes uncontrolled cell growth and survival, which are hallmark features of cancer [10], [11] Moreover, ERK1/2 signaling can crosstalk with other signaling pathways implicated in cancer, thereby amplifying oncogenic signaling networks. For example, ERK1/2 interacts with the PI3K-Akt pathway leading to increased cell proliferation and survival. Additionally, ERK1/2 signaling can influence the expression of genes involved in epithelial-mesenchymal transition (EMT), a process associated with cancer metastasis [12], [13]. As ERK1/2 signaling plays a central role in cancer, targeting this pathway has been the focus of cancer therapy. Small-molecule inhibitors targeting key components of the ERK1/2 pathway, such as Raf and MAPK/ERK kinase (MEK), have been developed and are being evaluated in clinical trials for various cancer types. Because PLX3397 targets upstream regulators of ERK1/2 signaling, such as c-Kit and CSF1R, it may also have therapeutic potential in certain cancers by indirectly modulating ERK1/2 activity. Based on the WebGestalt database, among the 389 genes related to the ERK1 and ERK2 cascades, only 16 genes were related to PLX3397. FLT3 is one of the genes related to the ERK signaling pathway, and PLX3397, which targets FLT3, may indirectly inhibit the ERK1/2 signaling pathway, which plays a key role in cancer development and progression (Table 3).

## Gene Ontology Molecular Function:

**Figure 4:**
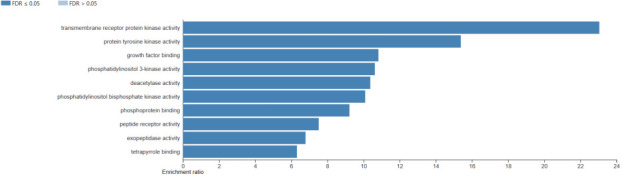
The bar shows the results of the analysis for molecular function affected with PLX3397

PLX3397 targets various molecules and pathways involved in cancer development and progression. Transmembrane receptor protein kinase and phosphatidylinositol 3-kinase activities are examples of molecular processes that PLX3397 might influence (Figure 4).

Transmembrane receptor protein kinase activity refers to the ability of receptor tyrosine kinase proteins (RTKs) to transfer signals across the cell membrane from outside to inside. These proteins typically consist of both extracellular and intracellular domains. The phosphorylation of intracellular domains triggers a series of downstream signaling events within the cell that regulate processes such as cell growth, differentiation, and survival, which are essential for various physiological processes. Approximately 99 genes control transmembrane receptor protein kinase activity, and only 15 genes could be targeted by PLX3397, and FLT3 is one of these genes (Table 4). FLT3 encodes Fms-like tyrosine kinase 3 (FLT3), a transmembrane receptor protein kinase primarily expressed on the surface of hematopoietic stem and progenitor cells in the bone marrow and is involved in cell survival, proliferation, and differentiation. Mutations in FLT3, such as internal tandem duplications (ITDs) and point mutations in the tyrosine kinase domain, are frequently found in acute myeloid leukemia (AML). These mutations lead to constitutive activation of FLT3 signaling, promoting uncontrolled growth and survival of leukemia cells [14].

Phosphatidylinositol 3-kinase (PI3K) activity is a critical component of the intracellular signaling pathways involved in regulating various cellular processes, including cell growth, survival, proliferation, metabolism, and motility. PI3K catalyzes the phosphorylation of phosphatidylinositol (PI) lipids to generate phosphatidylinositol 3,4,5-trisphosphate (PIP3). PIP3 activates downstream signaling proteins, which, in turn, regulate multiple cellular functions. Abnormal activation of PI3K signaling promotes uncontrolled growth and progression of cancer. Therefore, targeting PI3K activity has emerged as a promising strategy for cancer treatment [15]. Phosphatidylinositol 3-kinase activity is regulated by 99 genes, of which only seven can be targeted by PLX3397. FLT3 is one of the genes that control phosphatidylinositol 3-kinase activity (Table 5). FLT3 is frequently mutated and dysregulated in acute myeloid leukemia (AML), particularly through internal tandem duplication (ITD) mutations. FLT3-ITD mutations stimulate FLT3 kinase activity, which in turn activates downstream signaling pathways. Dysregulated signaling promotes cell proliferation, survival, and resistance to apoptosis, contributing to the pathogenesis and progression of AML. Targeting FLT3 by PLX3397 could interfere with phosphatidylinositol 3-kinase activity, which ultimately interferes with the development of malignancy.

To validate the efficacy of PLX3397 as an inhibitor of FLT3, computational studies, including docking, molecular dynamics simulation, and ADMET prediction, were performed. These experiments assessed binding affinity and identified key interactions between PLX3397 and FLT3. A crystallized structure of the FLT3 protein with a co-crystalized ligand, quizartinib (AC220), was obtained from the Protein Data Bank (PDB: 4XUF). Quizartinib (AC220) is an inhibitor that binds to the ATP-binding pocket of the FLT3 kinase domain in both wild type and mutated FLT3 [16].

## Ligand and protein preparation and molecular docking:

**Figure 5:**
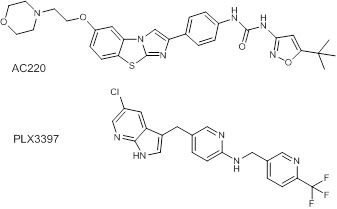
Chemical structures of native inhibitors (AC220) and PLX3397.

PLX3397 and a co-crystallized ligand (AC220) were prepared for docking, where energy-minimized 3D structures were generated and all possible ionization and tautomeric states were created. Both PLX3397 and the co-crystallized ligand (AC220) showed two isomers after they were converted into 3D structures. For docking, the FLT3 crystal structure (PDB ID: 4XUF) was selected because of the similarity between the structure of PLX3397 and the co-crystallized ligand (AC220) (Figure 5).

**Figure 6:**
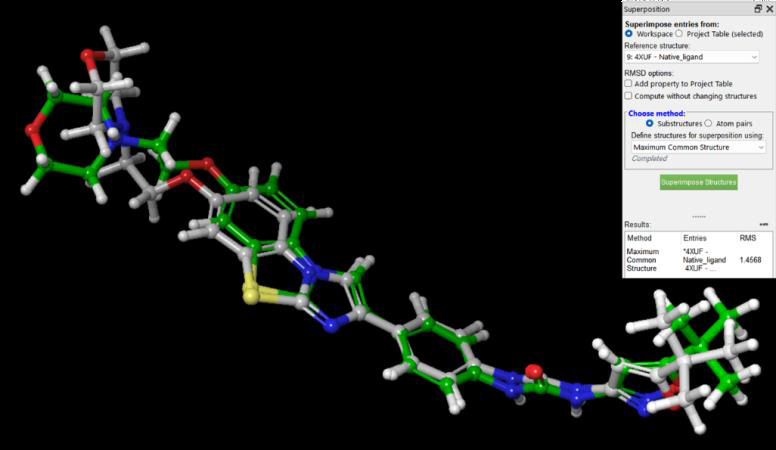
3D structure of re-docked AC220 (gray) superimposed on co-crystallized AC220 (green).

AC220 (1-(5-(tert-butyl)isoxazol-3-yl)-3-(4-(6-(2 morpholino ethoxy) benzo[d]imidazo [2,1-b]thiazol-2-yl) phenyl)urea ), consisting of aliphatic morpholinoethoxy bound to imidazobenzo-thiazole connected to tert-butyl-isoxazol-phenylurea. PLX3397 5-((5-chloro-1H-pyrrolo[2,3-b]pyridin-3-yl)methyl)-N-((6-(trifluoromethyl)pyridin-3-yl)methyl)pyridin-2-amine made of chloro-pyrrolopyridin bound to methylpyridin-amine that bound to trifluoromethylpyridin. Based on their structures, both AC220 and PLX3397 were expected to have similar 3D conformations in the binding pocket of the protein. The PDB file of the 4XUF crystal structure was downloaded from the Protein Data Bank (PDB), which was then prepared and minimized using Schrödinger's Protein Preparation Wizard. The docking process started with the definition of the grid box around the co-crystallized ligand to determine the docking location using the receptor-grid-generation tool in Maestro Schrödinger. To validate the accuracy of the docking method, re-docking of the co-crystallized ligand AC220 was performed back into the prepared protein. The primary goal of re-docking was to evaluate the accuracy of the predicted binding pose by comparing it to the crystallographic pose of AC220. The crystallographic pose and predicted binding pose were similar, with a root mean square deviation (RMSD) value of 1.4568. This finding indicates agreement between the predicted and observed binding interactions of AC220 (Figure 6).

After docking validation, docking of the 3D structures of AC220 and PLX3397 was performed using the extra precision (XP) mode. Docking produced derivatives that were ranked based on their score and approximated the free energy of binding; the more negative the value, the stronger the binding. The ranking depends on different docking scores, including gscore (best for ranking different compounds), emodel (best for ranking conformers), and XP gscore. Table 6 displays the docking scores of AC220 and its isomer, and PLX3397 and its isomer. Based on the glide gscores that sort docked compounds according to their poses, the first PLX3397 isomer showed a better gscore (-15.313 kcal/mol) than the first isomer of the native reference AC220 (-9.830 kcal/mol). Because of the docking scores of these two isomers, they were used for further computational studies.

**Figure 7:**
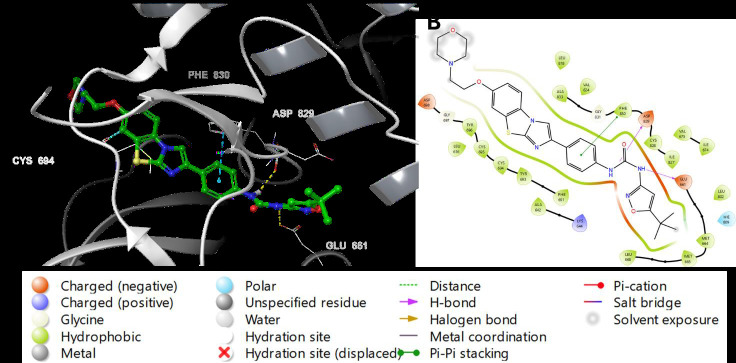
Binding mode of the co-crystallized ligand AC220 in the active site of FLT3 (PDB ID: 4XUF). AC220 is shown as a greenstick, whereas hydrogen bonds and ionic bonds are represented by yellow and blue dotted lines, respectively. (A)3D representation of FLT3 complexed with AC220 and (B)2D depiction.

**Figure 8:**
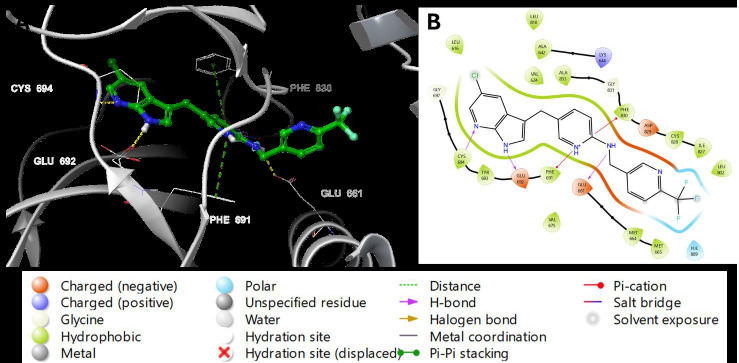
Binding mode of PLX3397 to the active site of FLT3 (PDB ID: 4XUF). PLX3397 is shown as green sticks, while hydrogen bonds and ionic bonds are represented by yellow and blue dotted lines, respectively. (A) 3D representation of FLT3 complexed with PLX3397 and (B) 2D depiction

**Figure 9:**
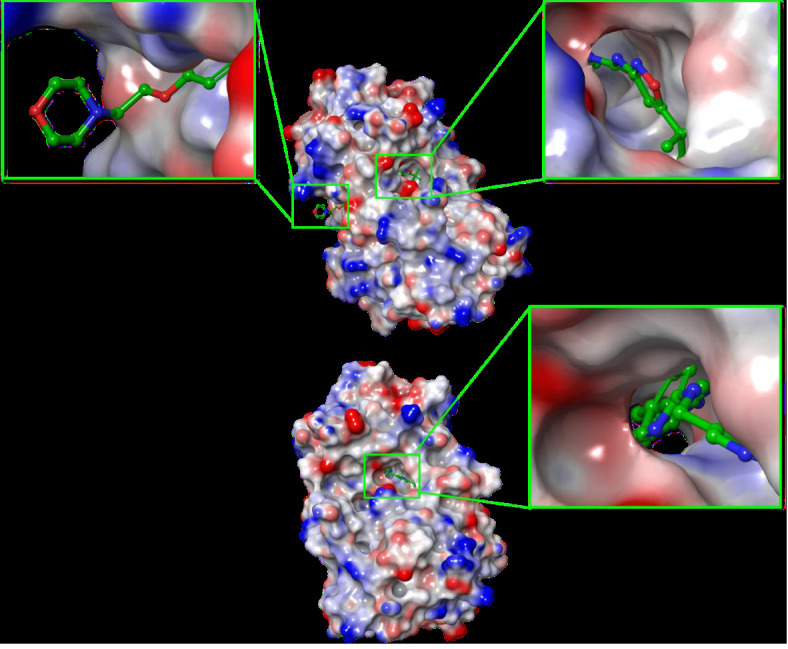
(A) Molecular surface display with an electrostatic potential color scheme for FLT3 complexed with the co-crystallized ligand AC220 after re-docking. (B) Molecular surface display with an electrostatic potential color scheme for FLT3 complexed with PLX3397 after docking.

The 3D docking representation revealed that both PLX3397 and AC220 H-bonded with Cys-694 and Glu-661. Phe-830 residue interacts with the aniline of AC220 via π-π stacking and with the middle pyridine of PLX3397 via π-cation stacking. Furthermore, the middle pyridine of PLX3397 forms another π-cation stacking with Phe-691, which explains its high docking score compared to that of AC220. The H-bonding of aniline in AC220 with the backbone carboxyl of Asp-829 corresponds to the H-bonding of the nitrogen of pyrrol in PLX3397 with the side chain carboxyl of Glu-692 (Figure 7A and Figure 8A). The 2D depictions of the binding modes of PLX3397 and AC220 were similar to those of the 3D depictions, with some differences. Glu-661, Glu-692, and Cys-694 form H-bonds with both PLX3397 and AC220. Phe-691 interacted through π-π stacking with the aniline of AC220 and with the middle pyridine of PLX3397. Phe-830 form pi-pi stacking with aniline ring while Asp-829 and Glu-661 interact with (tert-butyl) isoxazol)-phenylurea by hydrogen bonds (Figure 7B & Figure 8B). The molecular surface displayed in Figure B9 shows that PLX3397 occupied the binding pocket of the crystal structure. The co-crystallized ligand (AC220) seemed to have similar interactions with the protein; however, morpholine at the end of the chain did not occupy the distant pocket and remained exposed to the solvent (Figure 9A).

## Molecular dynamic simulation:

**Figure 10:**
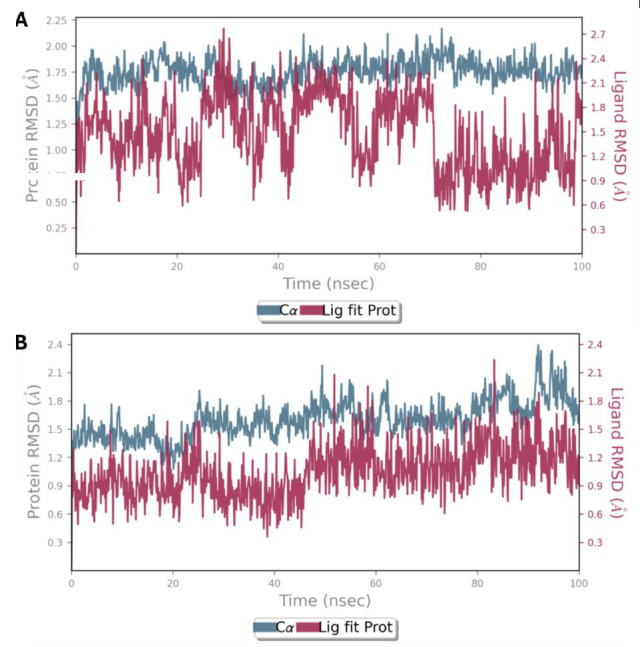
RMSD plot for (A) native ligand (AC220) and (B) PLX3397 with FLT3 (PDB ID: 4XUF) during the MD simulation.

MD simulation is a powerful computational technique used to study the movement and interactions of ligands and proteins over time. It is useful to understand the dynamics of protein-ligand complex stability under various conditions, such as changes in temperature, pressure, or chemical environment. Desmond software was used to perform MD studies on PLX3397 and native ligand (AC220) isomers with the best docking scores. (MD) simulation study, where complex structures were optimized under specific pH conditions (ranging from 7.0 to 2.0) followed by a simulation period of 100 nanoseconds (ns) to observe the behavior and convergence of system properties. The MD simulation was run in which protein-ligand complex structures were optimized under specific pH conditions (ranging from 7.0 to 2.0), followed by a simulation period of 100 ns to observe the behavior and convergence of the system properties. Analyzing interaction maps and root mean square deviation (RMSD) plots provides valuable insights into the stability and dynamics of these protein-ligand complexes. The stability of the complexes during a 100 ns simulation with reference to the initial time point (0 ns) was estimated by plotting the Root Mean Square Deviation (RMSD) of the protein-ligand complex over time. RMSD values of FLT3 protein plotted on the left y-axis and ligands plotted on the right y-axis. PLX3397 and AC220 complexes exhibited minor fluctuations within an acceptable range of 1-3 Å, indicating their stability. However, the AC220 complex showed more fluctuations than the PLX3397 complex, which reflects the stability of PLX3397 in the binding pocket (Figure 10).

**Figure 11:**
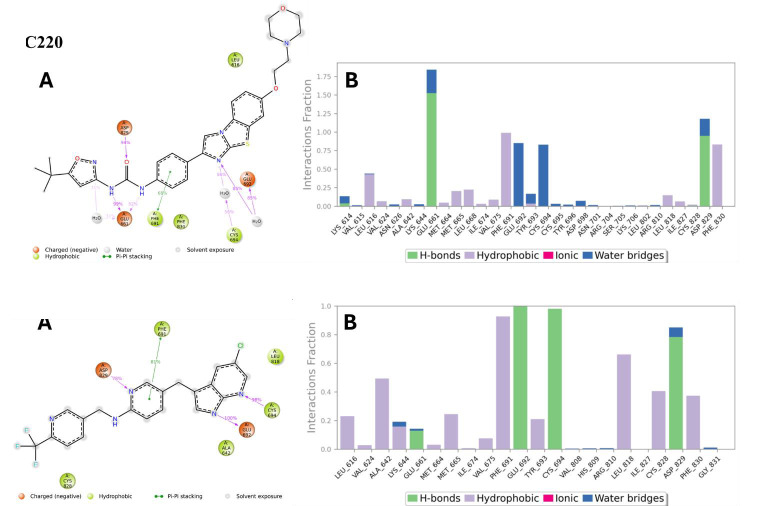
(A) Schematic diagram showing the detailed 2D atomic interactions of the reference ligands AC220 and PLX3397 with FLT3 that occurred over 30% of the simulation time in the selected trajectory. (B) Stacked-bar graph of FLT3 interactions with AC220 and PLX3397 throughout the simulation period.

The molecular interactions between the binding pocket amino acid residues and ligands that persisted for at least 30.0% of the simulation time within the selected frame (0.00 to 100.00 ns), as well as the docked poses that remained stable throughout the 100 ns simulation time, are displayed in Figure 11B. As shown in the top part of Figure 11B, Glu-661 formed direct H-bonds as well as water bridges with AC220 and had a normalized value of ~1.9, which indicates that the interactions were maintained for ~190% of the simulation time. A value >1 indicates a combination of more than one type of binding interaction. Other important interactions were Phe-691, Glu-692, Cys-694, Asp-829, and Phe-830, with values of approximately 1.0, 0.8, 0.8, 1.1, and 0.9, respectively. The bottom part of Figure 11B shows the key interactions of PLX3397 with Phe-691, Glu-692, Cys-694, Lue-818, and Asp-829, with values of ~0.9, ~1.0, ~1.0, ~0.7, and ~0.9, respectively. Figure 11A shows only the protein-ligand interactions that equal or exceed 30% of the simulation period. The top part of Figure 11A shows that Glu-661 interacts with AC220 via three binding types, including two H-bonds with di(azaneyl)methanone that existed for 99% and 52% of the simulation time and a water bridge 30%. Asp-829 H-bonded with the carbonyl of di(azaneyl)methanone continued for 94%, and Phe-691 formed pi-pi stacking with aniline held at 68%. Both Glu-692 and Cys-694 formed a water bridge with imidazo benzothiazole during 85% and 56% of the simulation period, respectively. The bottom part of Figure 11A shows that the residues interacted with PLX3397 for more than 30% of the simulation time. Cys-694 and Glu-692 H-bonded to terminal pyrrolopyridine were maintained at 99% and 100%, respectively. Phe-691 pi-pi staked and Asp-829 H-bonded with the middle pyridine for 81 and 78% of simulation time respectively.

## *In silico* ADMET Properties of PLX3397:

With Maestro's QikProp Schrödinger's module, the drug-likeness and ADMET characteristics of the PLX3397 isomer were predicted in terms of absorption, distribution, metabolism, excretion, and toxicity. The module can predict a wide range of physicochemical properties and other descriptors, including the number of reactive functional groups and possible metabolites, quickly and accurately. This allows the detection of compounds that may represent challenges in the later stages of drug discovery and development. Therefore, unnecessary experiments that will ultimately fail in clinical trials can be excluded. ADMET prediction evaluates the usefulness of PLX3397 isomers by identifying and assessing their drug-likeness, physicochemical properties, and anticipated toxicity profiles. For the PLX3397 isomers, several descriptors were predicted, and the majority of the ADMET descriptor predictions were within the recommended range or close to it. Table 7 presents the expected ADMET properties and descriptors.

*Recommended range: 95% of known drugs; #Stars: number of descriptors that fall outside the 95% range of the same values for known drugs. Large star numbers indicate less drug-likeness, and vice versa; dipole: computed dipole moment; SASA: Total solvent accessible surface area; DonorHB: estimated number H+ to be donated in HB; AcceptHB: estimated number H+ to be accepted in HB; QLogPo/w: predicted octanol/water partition coefficient; QPlogS: Predicted aqueous solubility; QPlogKhsa: Prediction of binding to human serum albumin; #Metab: number of possible metabolic reactions; QPlogBB: Predicted brain/blood partition coefficient; % Human Oral Absorption: Predicted human oral absorption on a 0 to 100% scale; QPlogHERG: Predicted IC50 value for blockage of HERG K+ channels; CNS: Predicted central nervous system activity; #RtvFG: Number of reactive functional groups. This computational study supports the predictions obtained from SwissTargetPrediction and SuperPred for the antitumor activity of PLX3397 through its interaction with FLT3. As FLT3 plays a critical role in AML pathogenesis, targeting FLT3 signalling represents a promising therapeutic strategy for the development of novel drugs. PLX3397 can be an indirect FLT3 inhibitor, and there has been some research interest in its potential application in acute myeloid leukemia (AML) because of its ability to target the tumor microenvironment by inhibiting CSF1R, which is involved in the regulation of macrophages and other immune cells within the bone marrow [17]. While PLX3397 primarily targets (CSF1R) and c-kit, it has been observed to have inhibitory effects on FLT3 signalling, particularly in cells with FLT3-ITD mutations [9].

## Conclusion:

FLT3 (Fms-like tyrosine kinase 3) is a protein that plays a role in cell growth and division. Mutations in FLT3 are commonly found in acute myeloid leukemia (AML), a type of cancer that affects the blood and bone marrow. PLX3397 is a small-molecule inhibitor that targets receptor tyrosine kinases, including CSF1R and KIT. It is being studied for its potential in the treatment of certain cancers, particularly that involving macrophage infiltration. In this study, *In silico* experiments, including molecular docking, molecular dynamics simulations, and ADMET prediction, were performed to determine the binding interaction of PLX3397 with FLT3. These studies intersect in cancer research, particularly in understanding the molecular mechanisms underlying AML, developing targeted therapies derived from PLX3398 against specific molecular targets, such as FLT3 mutations, and utilizing computational approaches (*In silico*) to accelerate drug discovery and optimize treatment strategies.

## Author contribution:

Conceptualization: RA; Methods and Data Analysis: RA; data curation and formal analysis: RA; writing-original draft, review and editing: RA. RA read and consented to the final version of the manuscript.

## Figures and Tables

**Table 1:** Top 30 protein targets of PLX3397 based on Swiss Target prediction analysis.

	**Target**	**Common name**	**Uniprot ID**	**ChEMBL ID**	**Target Class**	Probability*	**Known actives (3D/2D)**
1	Tyrosine-protein kinase receptor FLT3	FLT3	P36888	CHEMBL1974	Kinase	0.991386127	02-Jul
2	Cytochrome P450 2C9	CYP2C9	P11712	CHEMBL3397	Cytochrome P450	0.543083678	16-Feb
3	Cytochrome P450 2C19	CYP2C19	P33261	CHEMBL3622	Cytochrome P450	0.393972893	09-Jan
4	Cytochrome P450 3A4	CYP3A4	P08684	CHEMBL340	Cytochrome P450	0.192104608	29-Feb
5	Cytochrome P450 2D6	CYP2D6	P10635	CHEMBL289	Cytochrome P450	0	0 / 1
6	Cytochrome P450 1A2	CYP1A2	P05177	CHEMBL3356	Cytochrome P450	0	0 / 2
7	Histone deacetylase 3	HDAC3	O15379	CHEMBL1829	Eraser	0	22 / 0
8	Histone deacetylase 6	HDAC6	Q9UBN7	CHEMBL1865	Eraser	0	112 / 0
9	Histone deacetylase 4	HDAC4	P56524	CHEMBL3524	Eraser	0	155 / 0
10	Sodium channel protein type IX alpha subunit	SCN9A	Q15858	CHEMBL4296	Voltage-gated ion channel	0	45 / 0
11	Receptor-interacting serine/threonine-protein kinase 1	RIPK1	Q13546	CHEMBL5464	Kinase	0	16 / 0
12	Beta secretase 2	BACE2	Q9Y5Z0	CHEMBL2525	Protease	0	Nov-00
13	Insulin receptor	INSR	P06213	CHEMBL1981	Kinase	0	Aug-00
14	Tyrosine-protein kinase SRC	SRC	P12931	CHEMBL267	Kinase	0	18 / 0
15	Tyrosine-protein kinase HCK	HCK	P08631	CHEMBL3234	Kinase	0	Jul-00
16	Tyrosine-protein kinase Lyn	LYN	P07948	CHEMBL3905	Kinase	0	Nov-00
17	ORAI 1/2/3	ORAI1	Q96D31	CHEMBL2384891	Other ion channel	0	May-00
18	Tyrosine-protein kinase TIE-2	TEK	Q02763	CHEMBL4128	Kinase	0	30 / 0
19	Vascular endothelial growth factor receptor 1	FLT1	P17948	CHEMBL1868	Kinase	0	22 / 0
20	Platelet-derived growth factor receptor beta	PDGFRB	P09619	CHEMBL1913	Kinase	0	Oct-00
21	Platelet-derived growth factor receptor alpha	PDGFRA	P16234	CHEMBL2007	Kinase	0	Apr-00
22	Kinesin-1 heavy chain/ Tyrosine-protein kinase receptor RET	RET	P07949	CHEMBL2041	Kinase	0	Aug-00
23	C-C chemokine receptor type 1	CCR1	P32246	CHEMBL2413	Family A G protein-coupled receptor	0	36 / 0
24	Fibroblast growth factor receptor 3	FGFR3	P22607	CHEMBL2742	Kinase	0	116 / 0
25	Fibroblast growth factor receptor 1	FGFR1	P11362	CHEMBL3650	Kinase	0	Sep-00
26	Tyrosine-protein kinase BMX	BMX	P51813	CHEMBL3834	Kinase	0	Jan-00
27	Ephrin type-A receptor 3	EPHA3	P29320	CHEMBL4954	Kinase	0	Feb-00
28	Ephrin receptor	EPHB4	P54760	CHEMBL5147	Kinase	0	Sep-00
29	Tyrosine-protein kinase FES	FES	P07332	CHEMBL5455	Kinase	0	Jan-00
30	Lysyl-tRNA synthetase	KARS	Q15046	CHEMBL5575	Enzyme	0	Jan-00

**Table 2:** Top protein targets of PLX3397 based on SuperPred analysis

**Target Name**	**ChEMBL ID**	**UniProt ID**	**PDB Visualization**	**TTD ID**	**Min Activity**	**Assay Type**
Tyrosine-protein kinase receptor FLT3	CHEMBL1974	P36888	3QS9	Not Available	5 nm	IC50
Protein kinase C theta	CHEMBL3920	Q04759	5F9E	T23995	6 nm	Kd
Macrophage colony stimulating factor receptor	CHEMBL1844	P07333	4LIQ	T20333	10.8 nm	IC50
Stem cell growth factor receptor	CHEMBL1936	P10721	2EC8	T57700	27 nm	IC50

**Table 3:** ERK1 and ERK2 cascade genes targeted by PLX3397

**User ID**	**Gene Symbol**	**Gene Name**	**Entrez Gene**
FLT3	FLT3	fms related tyrosine kinase 3	2322
INSR	INSR	insulin receptor	3643
SRC	SRC	SRC proto-oncogene, non-receptor tyrosine kinase	6714
LYN	LYN	LYN proto-oncogene, Src family tyrosine kinase	4067
TEK	TEK	TEK receptor tyrosine kinase	7010
FLT1	FLT1	fms related tyrosine kinase 1	2321
PDGFRB	PDGFRB	platelet derived growth factor receptor beta	5159
PDGFRA	PDGFRA	platelet derived growth factor receptor alpha	5156
RET	RET	ret proto-oncogene	5979
CCR1	CCR1	C-C motif chemokine receptor 1	1230
FGFR3	FGFR3	fibroblast growth factor receptor 3	2261
FGFR1	FGFR1	fibroblast growth factor receptor 1	2260
EPHA3	EPHA3	EPH receptor A3	2042
EPHB4	EPHB4	EPH receptor B4	2050
KARS	KARS	lysyl-tRNA synthetase	3735
NTRK3	NTRK3	neurotrophic receptor tyrosine kinase 3	4916
IGF1R	IGF1R	insulin like growth factor 1 receptor	3480
HCRTR1	HCRTR1	hypocretin receptor 1	3061
FGFR2	FGFR2	fibroblast growth factor receptor 2	2263
ICAM1	ICAM1	intercellular adhesion molecule 1	3383
NPY5R	NPY5R	neuropeptide Y receptor Y5	4889
F2R	F2R	coagulation factor II thrombin receptor	2149
ABL1	ABL1	ABL proto-oncogene 1, non-receptor tyrosine kinase	25
MAP3K12	MAP3K12	mitogen-activated protein kinase kinase kinase 12	7786
GSTP1	GSTP1	glutathione S-transferase pi 1	2950

**Table 4:** Genes regulating transmembrane receptor protein kinase activity affected by PLX3397

**User ID**	**Gene Symbol**	**Gene Name**	**Entrez Gene**
FLT3	FLT3	fms related tyrosine kinase 3	2322
INSR	INSR	insulin receptor	3643
TEK	TEK	TEK receptor tyrosine kinase	7010
FLT1	FLT1	fms related tyrosine kinase 1	2321
PDGFRB	PDGFRB	platelet derived growth factor receptor beta	5159
PDGFRA	PDGFRA	platelet derived growth factor receptor alpha	5156
RET	RET	ret proto-oncogene	5979
FGFR3	FGFR3	fibroblast growth factor receptor 3	2261
FGFR1	FGFR1	fibroblast growth factor receptor 1	2260
EPHA3	EPHA3	EPH receptor A3	2042
EPHB4	EPHB4	EPH receptor B4	2050
NTRK3	NTRK3	neurotrophic receptor tyrosine kinase 3	4916
IGF1R	IGF1R	insulin like growth factor 1 receptor	3480
FGFR2	FGFR2	fibroblast growth factor receptor 2	2263
TGFBR1	TGFBR1	transforming growth factor beta receptor 1	7046

**Table 5:** Genes regulate phosphatidylinositol 3-kinase activity affected by PLX3397

**User ID**	**Gene Symbol**	**Gene Name**	**Entrez Gene**
**User ID**	**Gene Symbol**	**Gene Name**	**Entrez Gene ID**
FGFR1	FGFR1	fibroblast growth factor receptor 1	2260
FGFR2	FGFR2	fibroblast growth factor receptor 2	2263
FGFR3	FGFR3	fibroblast growth factor receptor 3	2261
FLT3	FLT3	fms related tyrosine kinase 3	2322
PDGFRA	PDGFRA	platelet derived growth factor receptor alpha	5156
PDGFRB	PDGFRB	platelet derived growth factor receptor beta	5159
SRC	SRC	SRC proto-oncogene, non-receptor tyrosine kinase	6714

**Table 6:** *In silico* docking results of PLX3397 and the co-crystallized ligand (AC220) with FLT3 (PDB: 4XUF)

**Title**	**Docking Score**	**XP GScore**	**Glide GScore**	**Glide Emodel**
PLX3397	-15.297	-15.313	-15.313	-86.772
PLX3397	-10.432	-12.583	-12.583	-77.621
AC220	-9.766	-9.83	-9.83	-126.381
AC220	-6.584	-7.933	-7.933	-128.69

**Table 7:** *In silico* ADMET Predicted properties of PLX3397 isomers.

**Molecule**	**Recommended range**	**PLX3397**	**PLX3397**
Mol_MW		417.82	417.82
#Stars	(0.0-5.0)	2	2
Dipole	(1-12.50)	7.822	7.558
SASA	(300-1000)	679.476	696.906
DonorHB	(0-6)	2	2
AccptHB	(2.0-20.0)	3.5	3.5
QPlogPo/w	(-2-6.5)	5.533	5.515
QPlogS	(-6.5-0.5)	-7.213	-7.525
QPlogHERG	Concern below -5	-6.293	-6.556
QPlogBB	(-3-1.2)	-0.216	-0.452
QPlogKp	(-1.5-1.5)	-1.613	-1.921
#Metab	(1-8)	5	5
QPlogKhsa	(-1.5-1.5)	0.862	0.902
%Human Oral Absorption	(<25% poor; >80% high)	100	100
CNS	(-2 inactive) (+2 active)	0	0
#RtvFG	(0-2)	0	0
